# Acute kidney injury: incidence, risk factors, and early outcomes in hospitalized children with sickle cell disease attending a tertiary hospital in Eastern Uganda

**DOI:** 10.1186/s12887-026-06893-5

**Published:** 2026-04-24

**Authors:** Asho warsame Mohamed, Hanan Asad Hassan, Grace Ndeezi, Iranezereje Dick, Abdullahi Abdirizak Farah, Sowdo Abdirizak Mohamed, Nasro Abdulkadir Omar, Muhiadin Omar Matan, Abdifatah Hersi Karshe, Fathi Ali Araye, Nur warsame Mahamed, Jama warsame Mohamed, Walyeldin Elfakey, Kumbowi Kumbakulu Patrick

**Affiliations:** 1 Department of Pediatrics and Child Health, Faculty of Clinical Medicine and Dentistry, Kampala International University, Ishaka, Uganda; 2 Department of psychiatric and mental health, Faculty of Clinical Medicine and Dentistry, Kampala International University, Ishaka, Uganda; 3 Department of Pediatrics, Faculty of Medicine, University of Bahri, Khartoum, Sudan; 4 Department of internal medicine, Faculty of clinical medicine and Dentistry, Kampala International University, Ishaka, Uganda; 5 Department of Human Resource Management, Faculty of Business Administration, Muhammad Ali Jinnah University Karachi (MAJU), Karachi, Pakistan; 6 Department of Public Administration and Management, Faculty of Management Studies, Islamic university in Uganda (IUIU), Mbale, Uganda

**Keywords:** Acute chest syndrome (ACS), Acute kidney injury (AKI), Hydroxyurea (HU), Non-steroidal anti-inflammatory drugs (NSAIDs), Sickle cell disease (SCD); Serum creatinine (Scr)

## Abstract

**Background:**

Many sickle cell disease (SCD) patients experience renal damage that impairs function, with acute kidney injury (AKI) significantly affecting both immediate and long-term outcomes. The only Ugandan study on this subject reported a high incidence and mortality of AKI among children with SCD, but did not assess other outcomes.

**Methods:**

This hospital-based prospective study was conducted between January and June 2024 at Jinja Regional Referral. AKI was diagnosed using KDIGO criteria, and participants were followed until discharge or death. Outcomes included length of hospital stay, mortality, and referral. Data were analyzed using SPSS v26, with Modified Poisson regression to identify significant risk factors (*P* < 0.05).

**Results:**

A total of 117 children were enrolled, slightly more females (52.1%) than males. Most were under 10 years, with 40.2% below 5 years and 42.7% aged 5–9 years? AKI occurred in 30 children, an incidence proportion of 25.6%. Independent risk factors for AKI included older age (aRR = 1.416), not using hydroxyurea (aRR = 1.628**)**, frequent Nonsteroidal Anti Inflammatory Drugs (aRR = 1.186) use for pain, presence of other illnesses (aRR = 1.995**)**, more than one transfusion in the preceding 6 months (aRR = 1.540), and stunting (aRR = 1.204) (*P* < 0.05 for all). Overall mortality was 5.1% (6/117), all occurring in children with AKI, giving a mortality of 20% in this group (*P* < 0.001). Prolonged hospital stay was significantly more common in the AKI group (54.5% vs. 13.8%, *P* < 0.001).

**Conclusions:**

The incidence of AKI was high, affecting one quarter of participants. Routine AKI screening is recommended for all SCD admissions. Ensuring hydroxyurea availability, educating patients on safe analgesic use, and close monitoring of SCD children with AKI could reduce mortality.

## Introduction

Sickle cell nephropathy (SCN) refers to chronic kidney disease (CKD) resulting from recurrent episodes of red blood cell sickling in individuals with sickle cell disease (SCD), These episodes can be either silent or present as painful vaso-occlusive crises, Some of these sickling episodes are silent, while others are painful vaso-occlusive crises [1].

The pathophysiology involves renal medullary hypoxia, acidosis, hyperosmolarity, hyposthenuria, hem concentration, and endothelial adhesion, leading to recurrent ischemia–reperfusion injury and hemoglobin polymerization [2]. AKI can significantly cause both immediate and long-term effects. Hospitalizations exacerbated by AKI have been linked to a higher risk of inpatient mortality, longer hospital stays, higher admission costs, and unfavorable outcomes for patients requiring intensive care unit (ICU)-level treatment in adults with SCD [2].

Globally, 300,000 children are born with SCA annually with 80% of affected children residing in Africa [3]. Migration from high-prevalence countries is contributing to the rise in SCD prevalence in industrialized nations [4]. In the UK over 14,000 people with SCD were estimated, whereas nations like Italy and Germany have more numbers due to an increase in the number of persons from Africa [5, 6].

In Africa, Sickle cell disease is a major cause of death for children under five in Africa. According to estimates of the worldwide burden, SCD is responsible for 6.4% of all under-5 deaths in Africa [7]. In Uganda, SCD is estimated to contribute to as much as 15% of deaths among children under five, particularly in countries with relatively low overall childhood mortality but high frequencies of the sickle cell allele [8].

In high-income countries (HIC), between 2.5 and 17% of children hospitalized with a vaso-occlusive pain crisis (VOC) develop AKI, making VOC a prevalent complication in children with SCA and a risk factor for AKI [3]. Risk factors for AKI in children with VOC include hypovolemia, and exposure to nephrotoxic medications, including non-steroidal anti-inflammatory drugs for pain management and aminoglycosides for the management of suspected infections [3].

as a previous study in Uguanda that involved 185 SCA children showed an incidence of 23.4% with a mortality rate of 7.5% vs. 0.9% in the non SCD [3].

In Uganda, SCD prevalence is estimated at 0.7–1% in the general population and up to 20% in some high-burden regions [9]. Local data on renal complications remain scarce, but evidence suggests children with SCD may be more vulnerable to acute kidney injury (AKI) due to reduced renal reserve, recurrent infections, dehydration, and delayed healthcare access [10].

AKI is a sudden decline in renal function, diagnosed by the Kidney Disease: Improving Global Outcomes (KDIGO) criteria as either a 1.5-fold rise in serum creatinine from baseline, a ≥ 0.3 mg/dL increase in serum creatinine within 48 h, or a reduction in urine output [3]. Both AKI and CKD are linked to increased inpatient mortality, prolonged hospitalization, and higher healthcare costs. Globally, SCD patients experience a 2–3 times higher incidence of AKI compared to the general population [11].

There is limited evidence from regional hospitals such as JRRH, where the incidence, risk factors, and outcomes of AKI may differ from those reported in national referral centers or high-income settings due to variations in healthcare resources, patient characteristics, and treatment access. Generating context-specific data from JRRH is therefore crucial to guide targeted prevention, strengthen early detection, and improve the management of AKI among children with SCD in resource-limited settings. This study aimed to determine the incidence, risk factors and early outcomes for acute kidney injury among hospitalized children with sickle cell disease at Jinja Regional Referral Hospital (JRRH).

## Methodology

### Study design

This was a hospital-based study that employed a prospective study design.

### Study setting

This study was conducted at Jinja Regional Referral Hospital (JRRH), located in Jinja City, in the Eastern Region of Uganda. JRRH is a government-funded tertiary healthcare facility that serves as the main referral center for the Busoga sub-region, which encompasses over 11 districts. It provides both primary and specialized medical care and serves a catchment population of approximately 3.5 million people. Jinja Regional Referral Hospital (JRRH) serves a predominantly rural population characterized by lower socioeconomic status, limited access to specialized care, and frequent stock-outs of essential medications such as hydroxyurea and blood products. Unlike national referral settings, patients at JRRH often present late with advanced disease due to financial constraints, reliance on traditional medicine, and limited health-seeking behavior. Additionally, high burden of infectious diseases such as malaria and sepsis, which are known contributors to acute kidney injury, is more pronounced in this setting. These contextual differences may significantly influence the incidence, risk factors, and outcomes of AKI among children with SCD, hence the need for a localized study.

### Eligibility criteria

#### Inclusion criteria

All children between the age of 1–12 years with sickle cell disease who were admitted to the pediatric department of JRRH, and their mother or next of kin consented to participate in the study.

#### Exclusion criteria

Children with structural kidney abnormalities (e.g., obstructive uropathy, congenital renal agenesis), Children with preexisting chronic kidney disease, Children missing baseline creatinine or where admission creatinine could not be obtained.

### Study participants

All children between the age of 1–12 years with sickle cell disease with in the catchment area of JRRH, who presented to the study center during the study period and fulfilled the eligibility criteria.

### Sample size

The sample size was calculated using Daniel’s formula:

$$n=\frac{z^2pq}{d^2}$$

Using findings by (Batte et al., [3]) at Mulago who reported that mortality occurred in 7.5% of the children with SCD and AKI, *P* = 0.075, d = 0.05 and Z = 1.96 for 95% level of significance. Substituting, *n* = 107.

On adding 10% to cater for loss of follow up, the sample size required was 117.

### Sampling technique and recruitment procedure

Consecutive enrolment for all the eligible participants was done till the sample size was attained. If the child was found to satisfy the eligibility criteria, their mother or guardian was explained to the purpose of the study and subsequently asked to consent. After consenting, they were enrolled for the study. The children were then be followed up.

### Study procedure

Children with sickle cell disease (SCD) admitted to the hospital were screened for eligibility, and consent was obtained from parents/guardians, with assent from children older than 7 years. After enrollment, socio-demographic and clinical data were collected through history and examination. Blood samples were taken at admission and every 48 h to measure serum creatinine, while urine output was monitored daily.

Acute kidney injury (AKI) was assessed using KDIGO criteria, defined by a rise in serum creatinine (≥ 0.3 mg/dl within 48 h or ≥ 1.5 times baseline within 7 days) or reduced urine output (< 0.5 ml/kg/hr for 6 h). AKI was staged into three levels based on the severity of creatinine increase. Patients were followed until discharge or death, and outcomes such as length of hospital stay, mortality, and referrals were recorded.

### Data collection instrument

A pre-tested, structured questionnaire administered by the investigator was used to document the sociodemographic and clinical characteristics of the study participants.

### Data management

The principal investigator kept questionnaires in lockable cabinets. Verification of data for completeness was done manually by the PI. Using an Excel data sheet, the data was entered and then exported to SPSS version 26 for additional analysis. To determine the amounts of missing values, significant outliers, independence of errors, multi-collinearity, and normalcy, exploratory data analysis was conducted. A computer with password protection was used to store data in order to prevent access by unauthorized individuals. Only approved individuals had access to the data as necessary.

### Ethical clearance

**Fig. 1:**
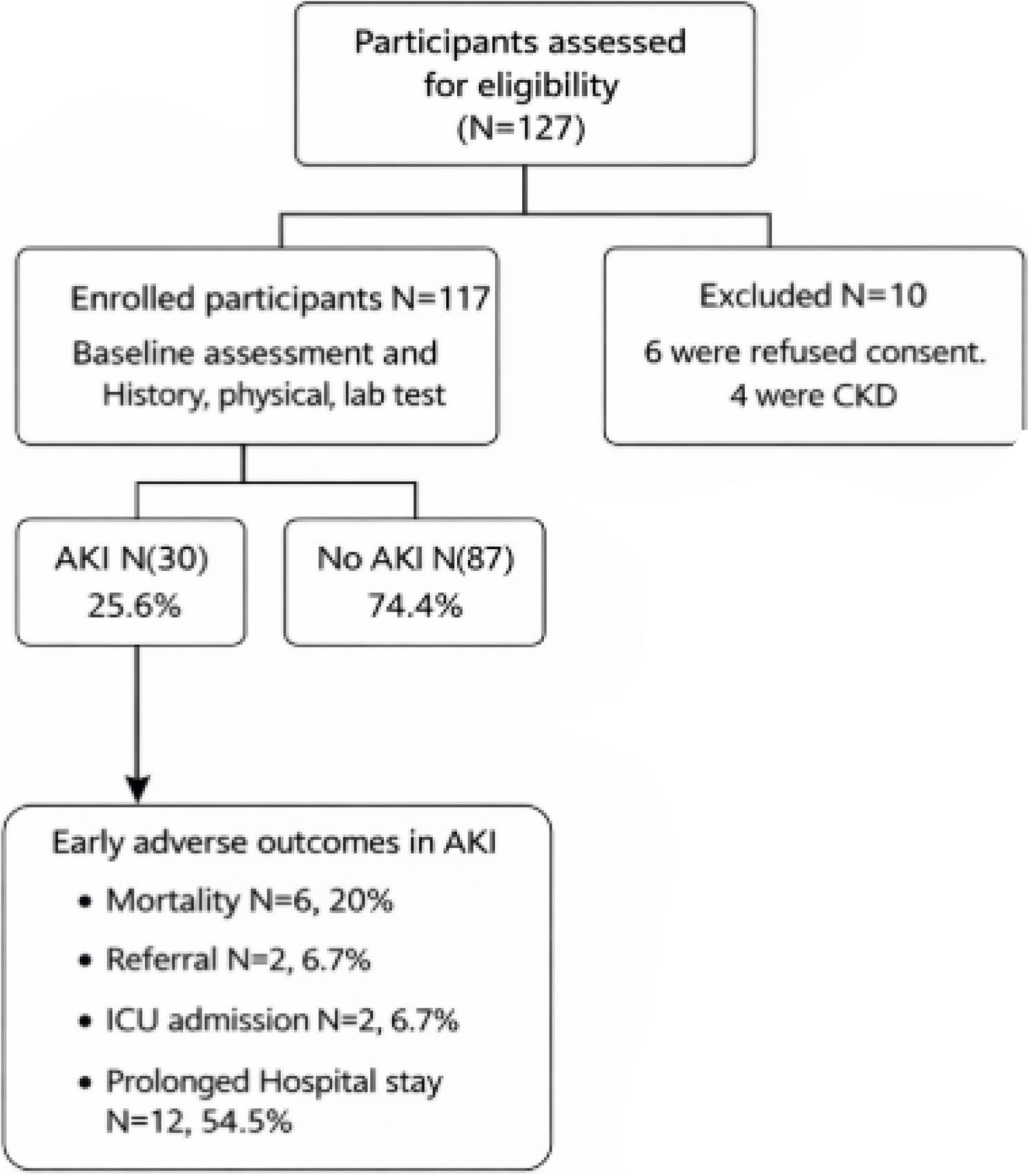
Study result flow chart

Before collecting any data, the principal investigator obtained ethical clearance from ethical review board of Kampala International University (KIU-REC-808). The principal investigator then obtained a permission letter from the Hospital director of JRRH and the respective in charge of the pediatric ward before starting data collection and written informed consent was obtained from parents/guardians (Fig. 1).

## Result presentation

### Study result chart

#### Baseline characteristics of the study participants

In this study, we enrolled 117 children, with slightly more females (52.1%) than males. Majority were aged below 10 years with 40.2% below 5 years and 42.7% aged between 5 and 9 years. Majority were from rural areas (73.5%). Only 47.0% were taking hydroxyurea. Majority took their medication in time as prescribed (94.9%). More than two thirds (69.2%) had had more than one admission in the previous 6 months, while in the same time period, 59.0% had received more than one blood transfusion. The details are shown in Table 1 below.

**Table 1:** Baseline characteristics of study participants

Characteristic	Frequency	Percentage
Sex		
Male	56	47.9
Female	61	52.1
Age category		
< 5	47	40.2
5–9	50	42.7
10+	20	17.1
Residence		
Urban	31	26.5
Rural	86	73.5
Education (father)		
None	18	15.4
Primary	28	23.9
Secondary	60	51.3
Tertiary	11	9.4
Education (mother)		
None	12	10.3
Primary	44	37.6
Secondary	50	42.7
Tertiary	11	9.4
Hydroxyurea use		
No	62	53.0
Yes	55	47.0
Pen V use		
No	66	56.4
Yes	51	43.6
NSAID use		
No	39	33.3
Yes	78	66.7
Exclusively BF		
No	25	21.4
Yes	92	78.6
Specific co-morbidities		
None	73	62.4
Malaria	20	17.1
Pneumonia	4	3.4
Others	20	17.1
Immunized up-to-date		
No	4	3.4
Yes	113	96.6
Take meds timely		
No	6	5.1
Yes	111	94.9
Admissions in the last 6 months		
≤ 1	36	30.8
2+	81	69.2
Transfusions in the last 6 months		
≤ 1	48	41.0
2+	69	59.0
Haemoglobin (g/dl)		
< 7	38	32.5
7+	79	67.5
Underweight		
No	101	86.3
Yes	16	13.7
Stunting		
No	87	74.4
Yes	30	25.6
Wasting		
No	103	88.0
Yes	14	12.0

*NSAID*  non-steroidal anti-inflammatory drugs, *BF *Breast feeding

### Incidence of acute kidney injury among hospitalized children with sickle cell disease

**Fig. 2:**
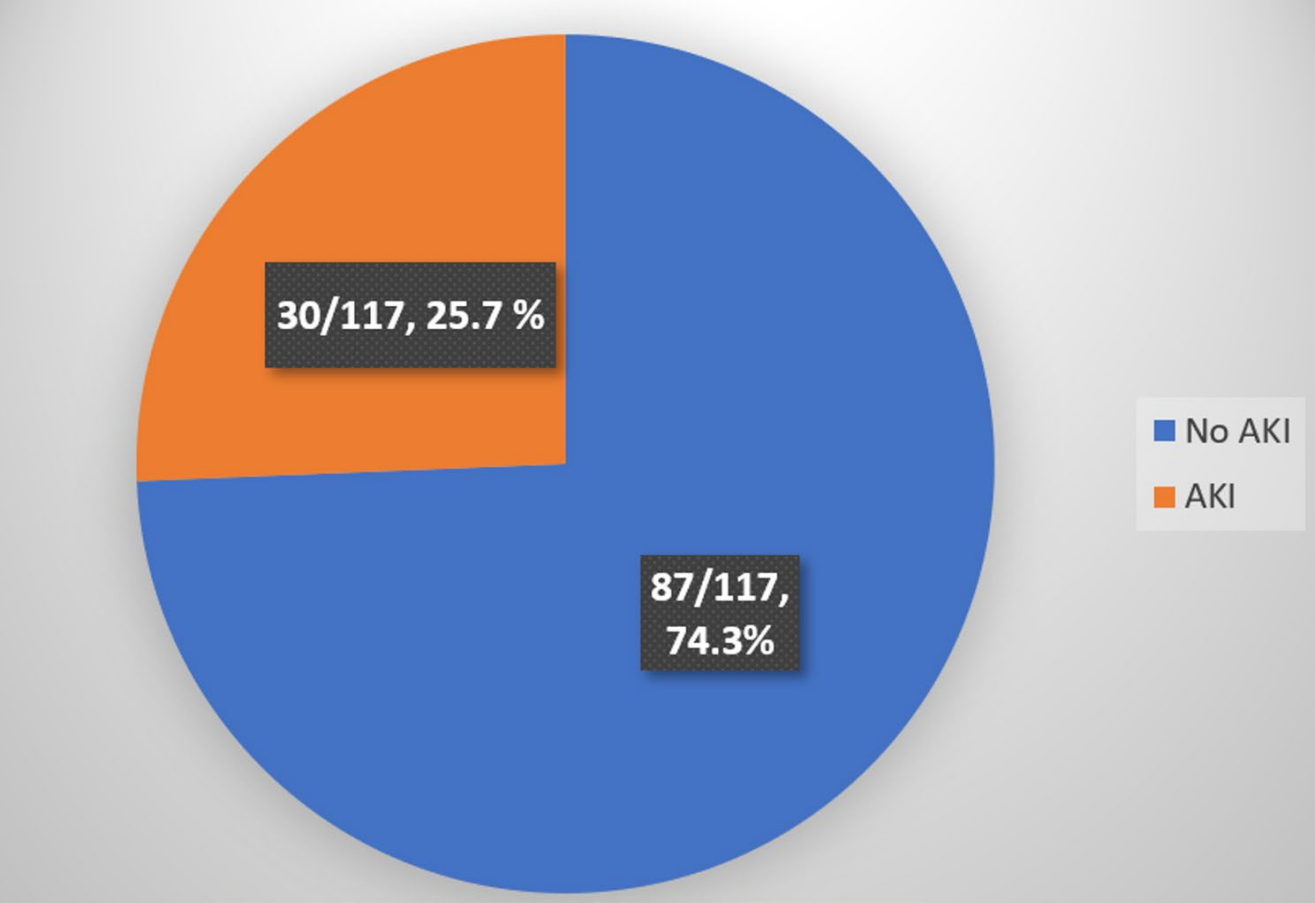
Incidence of acute kidney injury among hospitalized children with sickle cell disease. 87 out of 117(74.3%) were not having AKI, while 30 out of 117(25.7%) had AKI

Of the 117 participants enrolled, 30 had acute kidney injury, representing an incidence proportion of 25.6% with a 95% confidence interval of 18.0-33.3% (Fig. 2). Of the 30 that were confirmed to have AKI, one third (33.3%) had stage two, while the remaining two thirds had stage three.

### Risk factors of acute kidney injury among hospitalized children with sickle cell disease

The variables considered for multivariate analysis (*P* < 0.2) were: age category, residence, hydroxyurea use, pen V use, NSAID use and frequency, exclusive breast feeding, other illnesses and type, number of admissions and transfusions in the last 6 months, hemoglobin level and stunting as shown in Table 2 below.

**Table 2:** Bivariable analysis of risk factors of acute kidney injury among hospitalized children with sickle cell disease

Characteristic	No AKI, *N* = 87	AKI, *N* = 30	Bivariable analysis		
			cRR	95% CI	*P* value
Sex					
Male	40(46.0)	16(53.3)	1.058	0.903–1.240	0.487
Female	47(54.0)	14(46.7)	Ref		
Age category					
< 5	45(51.7)	2(6.7)	Ref		
5–9	36(41.4)	14(46.7)	1.268	1.105–1.454	**0.001**
10+	6(6.9)	14(46.7)	1.930	1.566–2.378	**< 0.001**
Residence					
Urban	27(31.0)	4(13.3)	Ref		
Rural	60(69.0)	26(86.7)	1.189	1.021–1.386	**0.026**
Birth order					
First born	32(36.8)	10(33.3)	Ref		
Second plus	55(63.2)	20(66.7)	1.029	0.874–1.211	0.731
Education (father)					
None	12(13.8)	6(20.0)	1.164	0.849–1.595	0.346
Primary	20(23.0)	8(26.7)	1.109	0.836–1.472	0.471
Secondary	46(52.9)	14(46.7)	1.053	0.818–1.354	0.688
Tertiary	9(10.3)	2(6.7)	Ref		
Education (mother)					
None	10(11.5)	2(6.7)	0.821	0.576–1.170	0.275
Primary	28(32.2)	16(53.3)	1.000	0.728–1.374	1.000
Secondary	42(48.3)	8(26.7)	0.816	0.603–1.103	0.286
Tertiary	7(8.0)	4(13.3)	Ref		
Hydroxyurea use					
No	36(41.4)	26(86.7)	1.414	1.229–1.628	**< 0.001**
Yes	51(58.6)	4(13.3)	Ref		
Pen V use					
No	46(52.9)	20(66.7)	1.113	0.953-1.300	**0.178**
Yes	41(47.1)	10(33.3)	Ref		
NSAID use					
No	37(42.5)	2(6.7)	Ref		
Yes	50(57.5)	28(93.3)	1.360	1.198–1.544	**< 0.001**
Exclusively BF					
No	15(17.2)	10(33.3)	1.200	0.973–1.480	**0.088**
Yes	72(82.8)	20(66.7)	Ref		
Co-morbidities					
None	69(79.3)	4(13.3)	Ref		
Malaria	14(16.1)	6(20.0)	1.278	1.038–1.573	**0.021**
Pneumonia	2(2.3)	2(6.7)	1.561	0.954–2.555	**0.077**
Others	2(2.3)	18(60.0)	2.328	2.021–2.682	**< 0.001**
Immunized uptodate					
No	2(2.3)	2(6.7)	1.287	0.783–2.114	0.319
Yes	85(97.7)	28(93.3)	Ref		
Take meds timely					
No	4(4.6)	2(6.7)	1.084	0.737–1.595	0.680
Yes	83(95.4)	28(93.3)	Ref		
Admissions in last 6/12					
≤ 1	32(36.8)	4(13.3)	Ref		
2+	55(63.2)	26(86.7)	1.234	1.068–1.425	**0.004**
Transfusions in last 6/12					
≤ 1	44(50.6)	4(13.3)	Ref		
2+	43(49.4)	26(86.7)	1.341	1.168–1.540	**< 0.001**
Haemoglobin (g/dl)					
< 7	20(23.0)	18(60.0)	Ref		
7+	67(77.0)	12(40.0)	1.380	1.155–1.647	**< 0.001**
Underweight					
No	73(83.9)	28(93.3)	Ref		
Yes	14(16.1)	2(6.7)	0.859	0.714–1.032	0.205
Stunting					
No	69(79.3)	18(60.0)	Ref		
Yes	18(20.7)	12(40.0)	1.213	1.098–1.474	**0.002**
Wasting					
No	77(88.5)	26(86.7)	Ref		
Yes	10(11.5)	4(13.3)	1.034	0.804–1.329	0.795

*cRR * Crude rate ratio, *CI* Confidence interval, *NSAIDS * non-steroidal anti-inflammatory drugs, *BF *Breast feeding

In the multivariable analysis, the independent risk factors of acute kidney injury among hospitalized children with sickle cell disease were: older age (aRR = 1.168, CI = 1.024–1.333, *P* = 0.021 for those aged 5–9 years and aaRR = 1.416, CI = 1.165–1.720, *P* < 0.001 for those older than 10 years), not using hydroxyurea (aRR = 1.128, CI = 1.016–1.252, *P* = 0.023), use of NSAIDs (aRR = 1.186, CI = 1.062–1.324, *P* = 0.002), specifically if used every time one feels pain (aRR = 1.261, CI = 1.095–1.453, *P* < 0.001), presence of other illnesses (aRR = 1.406, CI = 1.223–1.617, *P* < 0.001), more than one transfusion in the preceding 6 months (aRR = 1.201, CI = 1.073–1.346, *P* = 0.002), and presence of stunting (aRR = 1.204, CI = 1.090–1.376, *P* = 0.011). The rate of acute kidney injury was increased by 16.8% among those aged 5–9 years, by 41.6% among those aged 10 years and above, by 12.8% among those not using hydroxyurea, by 18.6% among those using NSAIDs, by 26.1% among those who take NSAIDs each time they feel any pain, by 40.6% among those with other illnesses, by 20.1% among those that received more than 1 transfusion in the preceding 6 months and by 20.4% among those who were stunted as detailed in Table 3 below.

**Table 3:** Multivariable analysis of risk factors of acute kidney injury among hospitalized children with sickle cell disease

Characteristic	Multivariable analysis		
	aRR	95% CI	*P* value
Age category			
< 5			
5–9	**1.168**	**1.024–1.333**	**0.021**
10+	**1.416**	**1.165–1.720**	**< 0.001**
Residence			
Urban			
Rural	1.109	0.821–1.284	0.126
Hydroxyurea use			
No	**1.128**	**1.016–1.252**	**0.023**
Yes			
Pen V use			
No	1.003	0.853-1.300	0.198
Yes			
NSAID use			
No			
Yes	**1.186**	**1.062–1.324**	**0.002**
NSAID use freq			
Never			
every time feels pain	**1.261**	**1.095–1.453**	**< 0.001**
some times when feels pain	1.137	0.883–1.290	0.251
Exclusively BF			
No	1.012	0.873–1.380	0.188
Yes			
Other illness			
No			
Yes	**1.406**	**1.223–1.617**	**< 0.001**
Specific illness			
None			
Malaria	1.278	0.738–1.673	0.061
Pneumonia	1.561	0.954–2.755	0.097
Others	1.828	0.921–2.912	0.051
Admissions in last 6/12			
≤ 1			
2+	1.134	0.868–1.325	0.054
Transfusions in last 6/12			
≤ 1			
2+	**1.201**	**1.073–1.346**	**0.002**
Hemoglobin (g/dl)			
< 7			
7+	1.080	0.855–1.347	0.061
Stunting			
No			
Yes	**1.204**	**1.090–1.376**	**0.011**

*aRR* adjusted rate ratio, *CI* Confidence interval

### Early adverse outcome of acute kidney injury among hospitalized children with sickle cell disease

Of the 117 children enrolled, mortality occurred in 6, representing an overall mortality of 5.1%. All the deaths occurred in the children with AKI, making the mortality in the AKI group to be 20%, which was significantly higher (*P* < 0.001) than that in those with no AKI. Only two children required ICU care and these were the only ones that were referred. These two children both had AKI. Excluding the children that passed away and they that were referred, prolonged hospital stay was seen in 20.5% of the participants. The proportion of children with prolonged hospital stay was significantly higher among those with AKI (54.5% versus 13.8%, *P* < 0.001). The details are shown in Table 4 below.

**Table 4:** Early adverse outcome of acute kidney injury among hospitalized children with sickle cell disease

Outcome	Overall (*N* = 117)	No AKI, *N* = 87	AKI, *N* = 30	*P* value
Mortality				< 0.001
Died	6(5.1)	0(0.0)	6(20.0)	
Survived	111(94.9)	87(100.0)	24(80.0)	
Timing of death (*N* = 6)				N/A
< 7days	2(33.3)	0(0.0)	2(33.3)	
> 8 days	4(66.7)	0(0.0)	4(66.7)	
Referred				0.064
No	115(98.3)	87(100.0)	28(93.3)	
Yes	2(1.7)	0(0.0)	2(6.7)	
ICU admitted				0.064
No	115(98.3)	87(100.0)	28(93.3)	
Yes	2(1.7)	0(0.0)	2(6.7)	
Prolonged stay for those discharged alive and not referred for ICU (*N* = 109)				< 0.001
≤ 7	85(72.6)	75(86.2)	10(45.5)	
8+	24(20.5)	12(13.8)	12(54.5)	

*ICU *intensive care unit, *AKI *acute kidney injury

## Discussion

This study was aimed at determining the incidence, risk factors and early outcomes for acute kidney injury among hospitalized children with sickle cell disease at Jinja regional referral hospital. The interpretation of our findings, what they add to the current practice and comparisons with previously published data is detailed in the following subsections stratified according to the specific objectives.

### Incidence of acute kidney injury among hospitalized children with sickle cell disease at Jinja regional referral hospital

Regarding the 117 participants enrolled, 30 had acute kidney injury, representing an incidence proportion of 25.7%. Of the 30 that were confirmed to have AKI, one third (33.3%) had stage two, while the remaining two thirds had stage three. This incidence was relatively high; and the exact causes cannot be clarified however some of the characteristics of the study participants such as not using hydroxyurea and use of NSAIDS among others could have contributed. A study conducted by [12] revealed that, hydroxyurea is a medication that increases fetal hemoglobin levels, which can reduce the number of sickled red blood cells and improve overall blood flow. A study conducted by [13] revealed that, in individuals with Sickle Cell Disease (SCD), NSAID use can increase the risk of Acute Kidney Injury (AKI) due to their impact on renal blood flow and the kidney’s ability to regulate itself. NSAIDs inhibit prostaglandin production, which can lead to vasoconstriction in the kidneys, reducing blood flow and potentially causing ischemic AKI. Additionally, NSAIDs can impair salt and water excretion, leading to fluid retention and further straining the kidneys. This is in agreement with our findings as 66.7% of the study participants used NSAIDS (Table 1), with 34.2% using every time they felt pain.

Comparable to our findings, A prospective study conducted in Uganda evaluated the prevalence and risk factors of acute kidney injury among 185 children aged 2–18 years with sickle cell anemia admitted for vaso-occlusive crisis. Kidney function was assessed at admission, after 24–48 h, and on day 7 or at discharge, with creatinine measured using an enzymatic method traceable to isotope-dilution mass spectrometry [3]. This proportion was similar to the one found in our study.

Comparable to our findings, to ascertain the effect of AKI in SCD, a two-year retrospective review was carried out in the USA and reported that 33% of the 197 admissions for vaso-occlusive pain crisis was linked to AKI [14]. Similarly, among infants with severe malaria or sickle cell disease evaluated in a prospective cohort study carried out in Uganda, a total of 185 sickle cell disease (SCD) children who were hospitalized due to a vaso-occlusive crisis were included in a study. According to the study, 23.2% of children with SCD had AKI at admission [15]. All these findings are comparable to our study since the proportions reported fall within the confidence interval of or findings (18.0-33.3%).

A retrospective study carried out in Saudi Arabia to assess renal outcomes in SCD patients who attended a hematology clinic at the National Guard Hospital in Jeddah reported that 29% of the patients, had renal imaging with abnormal results [16]. Similarly, to ascertain the prevalence and risk factors for renal dysfunction in children with sickle cell disease (SCD) aged 6 months to 12 years who were admitted to a tertiary hospital in Northwestern Tanzania, a cross-sectional hospital-based study reported that 48/153 (31.37%) children developed renal impairment [17]. These findings are also comparable to our study since the proportions reported fall within the confidence interval of or findings (18.0-33.3%).

A retrospective French study of 161 patients with sickle cell disease analyzed 254 vaso-occlusive events, including painful crises and acute chest syndrome. Acute kidney injury occurred in 4.3% overall, rising with severity from 2.3% in painful crises to 6.9% in moderate and 13.6% in severe acute chest syndrome [18]. The lower incidence may be due to the retrospective design, which could have missed some acute kidney injury cases, and the use of different diagnostic criteria, Acute Kidney Injury Network versus Kidney Disease (KIDIGO) vs. Acute Kidney Improving Global Outcomes (AKIN).

A retrospective United States study using Pediatric Health Information System data found that among pediatric sickle cell disease admissions for vaso-occlusive pain crisis, 2.5% were complicated by acute kidney injury, with 1.4% of patients experiencing at least one episode. The risk was higher among adolescents and young adults at first admission [19]. The lower incidence may be explained by the retrospective design, which could have missed some acute kidney injury cases, and the high-income setting, where better care for children with sickle cell disease may reduce the risk of complications.

### Risk factors of acute kidney injury among hospitalized children with sickle cell disease at Jinja regional referral hospital

Multivariable analysis identified older age, non-use of hydroxyurea, nonsteroidal anti-inflammatory drug use (especially with every pain episode), presence of comorbidities, more than one transfusion in the preceding 6 months, and stunting as independent risk factors for acute kidney injury. The risk increased by 16.8% in children aged 5–9 years and 41.6% in those ≥ 10 years, 12.8% among non-users of hydroxyurea, 18.6% with nonsteroidal anti-inflammatory drug use, 26.1% when used for every pain episode, 40.6% with comorbidities, 20.1% with > 1 transfusion, and 20.4% among stunted children.

AKI risk increased by 16.8% among children aged 5–9 years and by 41.6% among those aged 10 years and above. This is consistent with [19]. in the United States. Similarly, a systematic review by [20], reported older age as a significant predictor of kidney injury due to cumulative renal damage and reduced renal reserve over time. In Uganda [3], Increasing age was a consistent risk factor, likely due to cumulative kidney damage from repeated sickling episodes.

Children who were not using hydroxyurea had a 12.8% higher risk of developing AKI. Hydroxyurea increases fetal hemoglobin, reduces red blood cell sickling, and improves renal perfusion [21]. This is in agreement with [19], which reported Hydroxyurea as protective against AKI in pediatric SCD patients. In our cohort, 86.7% of children with acute kidney injury had never used hydroxyurea, highlighting limited access and its contribution to increased renal risk in Uganda (Table 2). A study conducted by [22], revealed that transfusion reactions, including delayed hemolytic transfusion reactions (DHTR), can cause hemolysis and release free hemoglobin, which can be toxic to the kidney, hence increasing the risk of AKI. This is in agreement with our findings where majority, 86.7% of the SCD patients that developed AKI were transfused more than once in a period of 6 months (Table 2).

Acute kidney injury (AKI) hospitalization rates are increasing, especially among older patients [23]. This is in agreement with our findings where almost half (46.7%) of the SCD patients that developed AKI were 10years and above (Table 2).

A study conducted by [24], revealed that Comorbid conditions, particularly involving the heart and lungs, increase the risk of acute kidney injury in sickle cell disease by worsening renal vulnerability. This aligns with our findings, where 86.7% of affected patients had other illnesses.

Furthermore, a study conducted by [25], revealed that Stunting, reflecting chronic malnutrition, may increase the risk of acute kidney injury in sickle cell disease by worsening renal vulnerability through electrolyte imbalances and reduced physiological reserve. This is consistent with our findings, where 40% of affected patients were stunted.

A United States retrospective study using Pediatric Health Information System data found that increasing age was associated with higher odds of acute kidney injury, while hydroxycarbamide use was protective [19]. Also comparable was, a systematic review which reported that increasing age was associated with AKI [22]. Also in agreement, a study in Uganda reported that AKI risk factors included increasing age and infections [3].

### Early adverse outcome of acute kidney injury among hospitalized children with sickle cell disease at Jinja regional referral hospital

Among 117 children, 6 died (5.1%), all with acute kidney injury, giving a mortality of 20% in this group. Two children required intensive care, both with acute kidney injury. Excluding deaths and referrals, prolonged hospital stay occurred in 20.5% overall and was significantly higher in those with acute kidney injury (54.5% vs. 13.8%). Consistent with our findings, a United States retrospective study showed that acute kidney injury in pediatric sickle cell disease was associated with higher intensive care admissions, longer hospital stays, increased costs, higher readmission rates, and more comorbidities [19]. Similarly, to ascertain the effect of AKI in SCD, a two-year retrospective review was carried out in the USA. The study revealed that patients with pain and AKI required longer hospitalizations than patients without AKI [14]. This is in agreement with our findings where majority (54.5%) of AKI individuals that had SCD had prolonged hospital stay (Table 4).

This study was limited by follow-up restricted to hospitalization, preventing assessment of long-term outcomes and post-discharge events. Additionally, structural causes of acute kidney injury were not systematically evaluated due to limited access to diagnostic imaging. The incidence of acute kidney injury among hospitalized children with sickle cell disease was high, affecting one quarter of participants. Independent risk factors included older age, non-use of hydroxyurea, nonsteroidal anti-inflammatory drug use, comorbid conditions, multiple transfusions, and stunting. Mortality and prolonged hospital stay were significantly higher among children with acute kidney injury. Routine screening for acute kidney injury should be implemented for all children admitted with sickle cell disease, with priority given to high-risk groups identified in this study. Improving access to hydroxyurea and promoting appropriate analgesic use may help reduce the risk of acute kidney injury and its associated complications.

## Data Availability

The data can be obtained upon request from the authors.

## Appendix I

### Measurement of Study Variables

Dependent Variables:

- **Age**: The length of time a person has lived, measured in complete years from the date of birth to the present date.
- **Gender**: The biological classification of individuals as male or female, based on reproductive anatomy and chromosomes.
- **Residence**: The geographic location or dwelling where a person lives, which can influence access to healthcare and resources.
- **Birth order**: The sequence or rank in which a child is born within a family, such as firstborn, middle, or lastborn.
- **Parental education level**: The highest formal education attained by a mother and/or father, often linked to socioeconomic status.

### Medical factors

- **Frequency of admissions**: The number of times a patient is hospitalized within a specified period, indicating disease severity or recurrence.
- **Recent transfusions**: The administration of blood or blood components to a patient within the recent past, usually weeks or months.
- **Hemoglobin level**: The concentration of hemoglobin in the blood, measured in grams per deciliter, reflecting oxygen-carrying capacity.
- **NSAIDs use**: The consumption of non-steroidal anti-inflammatory drugs for pain, inflammation, or fever control.
- **Hydroxyurea use**: The therapeutic use of hydroxyurea, commonly for sickle cell disease to reduce complications and improve blood parameters.
- **Drug adherence**: The degree to which a patient correctly follows medical prescriptions in timing, dosage, and duration.
- **Pen V use**: The regular intake of penicillin V, often as a preventive measure against bacterial infections in certain chronic conditions.
- **Immunization history**: A documented record of vaccines received throughout life, reflecting protection against specific diseases.
- **Exclusive breastfeeding**: Providing only breast milk to an infant, without any other liquids or solids, typically for the first six months of life.
- **Nutritional status**: The overall condition of health as influenced by the intake and utilization of nutrients.
- **Prolonged hospital**: A hospital stays exceeding the typical expected duration for a given illness or procedure.
- **Current medical illness**: Any disease or health condition that a person is presently experiencing and receiving treatment for.
- **Referral**: The act of directing a patient to another healthcare provider or facility for specialized assessment or management.
- **AKI**: Acute kidney injury, a sudden decline in kidney function leading to waste accumulation and fluid imbalance.
